# Identifying the Machine Translation Error Types with the Greatest Impact on Post-editing Effort

**DOI:** 10.3389/fpsyg.2017.01282

**Published:** 2017-08-02

**Authors:** Joke Daems, Sonia Vandepitte, Robert J. Hartsuiker, Lieve Macken

**Affiliations:** ^1^Department of Translation, Interpreting and Communication, Ghent University Ghent, Belgium; ^2^Department of Experimental Psychology, Ghent University Ghent, Belgium

**Keywords:** post-editing, machine translation, translation quality, post-editing effort, effort indicators

## Abstract

Translation Environment Tools make translators’ work easier by providing them with term lists, translation memories and machine translation output. Ideally, such tools automatically predict whether it is more effortful to post-edit than to translate from scratch, and determine whether or not to provide translators with machine translation output. Current machine translation quality estimation systems heavily rely on automatic metrics, even though they do not accurately capture actual post-editing effort. In addition, these systems do not take translator experience into account, even though novices’ translation processes are different from those of professional translators. In this paper, we report on the impact of machine translation errors on various types of post-editing effort indicators, for professional translators as well as student translators. We compare the impact of MT quality on a product effort indicator (HTER) with that on various process effort indicators. The translation and post-editing process of student translators and professional translators was logged with a combination of keystroke logging and eye-tracking, and the MT output was analyzed with a fine-grained translation quality assessment approach. We find that most post-editing effort indicators (product as well as process) are influenced by machine translation quality, but that different error types affect different post-editing effort indicators, confirming that a more fine-grained MT quality analysis is needed to correctly estimate actual post-editing effort. Coherence, meaning shifts, and structural issues are shown to be good indicators of post-editing effort. The additional impact of experience on these interactions between MT quality and post-editing effort is smaller than expected.

## Introduction

In order to improve Translation Environment Tools, we need to find objective ways to assess post-editing effort before presenting machine translation output to the translator. In current tools, it is often still the translator that has to decide whether or not the output provided by the machine translation system is worth post-editing, or whether it would be faster to simply translate from scratch (Offersgaard et al., 2008). Letting the post-editors make this decision, however, costs time and effort. It would be much more cost-efficient to have a system capable of pre-assessing MT suitability (Schütz, 2008; Denkowski and Lavie, 2012) by predicting post-editing effort. At the same time, it is still largely unclear how post-editing effort should be defined and measured and whether differences in effort between more and less experienced translators can be observed.

Current systems often make use of product analysis to evaluate post-editing effort, but the question remains whether these methods measure actual effort or not, as a product is a result of a process, not necessarily a reflection of the effort involved in the process itself. Process measures are therefore expected to give a more accurate indication of actual effort, as they measure the effort as it is taking place. When discussing effort, it is also important to take the factor ‘experience’ into account. Student translators have been shown to work differently compared to professional translators, and their attitude toward machine translation is also expected to be different.

In this study, we observe the impact of machine translation errors on different post-editing effort indicators: the often used product effort indicator human-targeted translation error rate (HTER), and some of the commonly used process effort indicators [fixation duration, number of fixations, (average) pause ratio, post-editing duration, and production units].

To collect the data, we set up a post-editing experiment with professional translators and student translators, using keystroke logging and eye-tracking. We first verify whether all effort indicators are influenced by machine translation quality, and then identify the specific types of machine translation errors that have the greatest impact on each of the effort indicators. This study is an extension and improvement of a previous study (Daems et al., 2015), in which only the student data was analyzed and only process effort indicators were observed.

It is hypothesized that, while all effort indicators are expected to be influenced by machine translation quality, the product effort indicator will be impacted by different types of errors than the process indicators, suggesting that product effort indicators in isolation are not a sufficiently accurate measure of actual post-editing effort. We expect there to be overlapping error types between the different process effort indicators, as they are to a certain extent related to one another (Krings, 2001). With regards to experience, it is hypothesized that different machine translation errors impact the effort indicators differently for student translators and for professional translators.

In the following sections, we will first discuss possible measures of post-editing effort, the influence of machine translation quality and the influence of experience.

### Related Research

Post-editing effort has been assessed via product analysis (by comparing machine translation output to a reference translation or post-edited sentence) and via process analysis (by observing aspects of the post-editing process, such as duration, production units, pauses, and fixation behavior). These measures will be discussed in more detail in the following paragraphs.

#### Assessing PE Effort via Product Analysis

Often used automatic metrics like BLEU (Papineni et al., 2002) or METEOR (Banerjee and Lavie, 2005) compare MT output to reference translations to evaluate a machine translation system’s performance. Whereas the values given by such metrics can be used to benchmark and improve MT systems, they are created on the basis of translations made independently of the MT output and thus do not necessarily provide post-editors with valid information about the effort that would be involved in post-editing the output. In addition, a score given by such metrics contains no information about the complexity of specific errors that need to be fixed during post-editing.

More recently, research into machine translation quality estimation (QE) has moved away from the independent reference translations, as used by automatic metrics, to reference post-edited sentences. Many of these QE systems have been trained on HTER data (Snover et al., 2006). HTER measures the edit distance between the MT output and a post-edited version of that MT output. The benefit of using HTER is the fact that it is relatively easy to apply, as it only requires MT output and a post-edited text. The underlying assumption when using HTER for QE is that HTER is an indication of actual post-editing effort (Specia and Farzindar, 2010), which implies that all edits made to MT output by a human are expected to require a comparable amount of effort. However, HTER has one important limitation: as HTER focuses on the final product, it does not take the actual process into account, so its relationship to post-editing effort is questionable. For example, a post-editor can return to the same phrase multiple times during the post-editing process, changing that particular phrase each time, but settling on one specific solution in the end. HTER will indicate how different this final solution is from the original MT output, but it does not take into account all edits made during the process. In addition, the number of edits required to solve an issue does not necessarily correlate with the cognitive demand.

#### Assessing PE Effort via Process Analysis

According to Krings (2001), there are three main types of process-based post-editing effort. Of these three, the easiest to define and measure is temporal effort: how much time does a post-editor need to turn machine translation output into a high quality translation? The second type of post-editing effort is somewhat harder to measure, namely technical effort. Technical effort includes all physical actions required to post-edit a text, such as deletions, insertions, and reordering. The final type of effort is cognitive effort. It refers to the mental processes and cognitive load in a translator’s mind during post-editing, and can, presumably, be measured via fixation data. While it is important to distinguish between these three types of post-editing effort conceptually, it must be noted that they are, to some extent, related to one another. Temporal effort is determined by a combination of cognitive effort and technical effort, and while an increase in technical effort does not necessarily correspond to an increase in cognitive effort, the technical process is still guided by cognitive processes.

Since effort can be defined in many different ways, researchers have used different methods to measure it. Koponen et al. (2012), for example, inquired into temporal and technical effort. They used a cognitively motivated MT error classification created by Temnikova (2010) and found that more time was needed to post-edit sentences that contained more cognitively demanding errors. They considered the number of keystrokes as well, but found no relationship between the number of keystrokes and the error types. Keystrokes were more strongly influenced by individual differences between participants.

A more direct cognitive measure was used by Jakobsen and Jensen (2008), who looked at fixation duration and the number of fixations during tasks of increasing complexity (from reading to translation). They found longer average fixation durations and a higher number of fixations as the complexity of a task increased. Doherty and O’Brien (2009) looked at the same variables, focusing specifically on the reading of MT output. They found a higher number of fixations when reading bad MT output than when reading good MT output, but they did not find a significant difference between the average fixation durations for both outputs.

Lastly, pauses have also been used a measure of effort. O’Brien (2006), for example, assumed that a higher number of negative translatability indicators (NTIs, i.e., elements in the source texts that are known to be problematic for MT) would be cognitively more demanding for a post-editor. She suggested pause ratio (total time in pauses divided by the total editing duration) as a possible indicator of cognitive effort, but she did not find conclusive evidence for a relationship between pause ratio and the number of NTIs in the source text.

Later, Lacruz et al. (2012) took the number of editing events in a sentence to be an indication of cognitive effort. They introduced the average pause ratio (the average duration per pause in a segment divided by the average duration per word in the segment) as an answer to O’Brien’s (2006) pause ratio, arguing that pause ratio is not sensitive enough as a measure for cognitive activity, as it does not take average pause length into account. While their results were promising, it must be noted that they reported on a case study with only one participant, and that they used a different measure of cognitive demand compared to O’Brien (2006).

#### Impact of MT Quality

Denkowski and Lavie (2012) made a clear distinction between analyzing MT as a final product and MT fit for post-editing, saying that evaluation methods for the first may not necessarily be appropriate for the latter. It is the latter that the present article will be concerned with: does MT quality have an impact on PE effort, and, if so, which kinds of MT errors have the highest impact on this effort?

The problem has been approached by using human quality ratings ranging from ‘good’ to ‘bad’ (Doherty and O’Brien, 2009; Koponen, 2012; Popovic et al., 2014) and error typologies (Koponen et al., 2012; Stymne et al., 2012).

Doherty and O’Brien (2009), Koponen (2012), and Popovic et al. (2014) used human-assigned sentence ratings with four or five levels, with the highest score indicating that no post-editing was needed and the lowest score indicating that it would be better to translate from scratch. Whereas Popovic et al. (2014) included MT output at all levels in their analysis, Doherty and O’Brien (2009) and Koponen (2012) limited theirs to the MT segments with highest and lowest quality. It is therefore hard to directly compare the three studies. For the lower quality sentences, Koponen (2012) and Popovic et al. (2014) found an increase in the number of word order edits and Doherty and O’Brien (2009) found a higher number of fixations.

Regarding error typologies, Koponen et al. (2012) used the classification proposed by Temnikova (2010), which contains various MT output errors ranked according to cognitive demand, and Stymne et al. (2012) used the classification proposed by Vilar et al. (2006). This difference in classification makes it hard to compare both studies, although they both found word order errors and incorrect words to impact post-editing effort the most: Koponen et al. (2012) studied their relationship with post-editing duration, and Stymne et al. (2012) studied their relationship with fixation duration.

For future work, researchers suggest using more fine-grained error typologies (Koponen et al., 2012; Stymne et al., 2012) and different languages (Koponen et al., 2012; Stymne et al., 2012; Popovic et al., 2014).

#### Impact of Translation Experience

The effort involved in post-editing can be expected to be different for professional translators and students on the basis of previous studies in the field of translation and revision research.

Inexperienced translators have been shown to treat the translation task as a mainly lexical task, whereas professional translators pay more attention to coherence and style (Tirkkonen-Condit, 1990; Séguinot, 1991). Students have also been shown to require more time (Tirkkonen-Condit, 1990), and a higher number of fixations and pauses than professional translators while translating (Dragsted, 2010).

A comparable trend is found in revision research. Sommers (1980), for example, found that experts adopt a non-linear strategy, focusing more on meaning and composition, whereas student revisers work on a sentence level and rarely ever reorder or add information. Hayes et al. (1987), too, reported that expert revisers first attend to the global structure of a text, whereas novice revisers attend to the surface level of a text. Revision is seen as a complex process that puts a great demand on working memory. Broekkamp and van den Bergh (1996) found that students were heavily influenced by textual cues during the revision process. For example, if a text contained many grammatical errors, the reviser’s focus switched to solving grammatical issues, and more global issues were ignored.

It remains to be seen whether these findings can be extrapolated to the post-editing process. The study by de Almeida and O’Brien (2010) is, to the best of our knowledge, the only one linking experience to the post-editing process. They found that more experienced translators were faster post-editors and made more essential changes as well as preferential changes.

### Hypotheses

In this article, we focus on the following research questions: (i) are all effort indicators influenced by machine translation quality, (ii) is the product effort indicator HTER influenced by different machine translation error types than the process effort indicators, (iii) is there an overlap between the error types that influence the different process effort indicators, and (iv) is the impact of machine translation error types on effort indicators different for student translators than for professional translators?

On the basis of the above-mentioned research, we expect that a decrease in machine translation quality will lead to an increase in post-editing effort, as expressed by an increase in HTER (Specia and Farzindar, 2010), the number of production units^1^ (Koponen, 2012; Popovic et al., 2014), the number of fixations (Doherty and O’Brien, 2009), post-editing time (Koponen et al., 2012), fixation duration (Stymne et al., 2012), pause ratio (O’Brien, 2006), and a decrease in average pause ratio (Lacruz et al., 2012).

As HTER is a product measure, we expect it to be influenced by different machine translation error types than the process effort indicators, which we expect to be influenced by comparable error types, as they are, to a certain extent, related (Krings, 2001). More specifically, we expect process effort indicators to be influenced most by mistranslations and word order issues (Koponen et al., 2012; Stymne et al., 2012) and lexical or semantic issues (Popovic et al., 2014).

Given the notion that inexperienced revisers focus more on grammatical errors when there is an abundance of grammatical errors (Broekkamp and van den Bergh, 1996), and the fact that student translators treat translation as a lexical task (Tirkkonen-Condit, 1990), we expect students to focus mostly on the grammatical and lexical issues, whereas professional translators are expected to pay more attention to coherence, meaning, and structural issues (Sommers, 1980). This means that we expect to see a greater increase in post-editing effort with students than with professional translators when there is an increase in grammatical and lexical issues in the text, and we expect a greater increase in post-editing effort with professional translators than with students when there is an increase in coherence, meaning, or structural issues.

## Materials and Methods

In order to collect both process and product data for translators with different levels of experience, we conducted a study with student and professional translators, registering the post-editing process by means of keystroke logging and eyetracking. As product effort indicator, we measured HTER. As process effort indicators, we measured fixation duration, number of fixations, (average) pause ratio, post-editing duration, and production units.

### Participants

Participants were 13 professional translators (3 male and 10 female) and 10 master’s students of translation (2 male and 8 female) at Ghent University who had passed their final English translation examination, meaning that they were ready to start working as professional translators. All participants were native speakers of Dutch. With the exception of one professional translator, who had 2 years of experience, all other translators had experience working as a full-time professional translator between 5 and 18 years. Students’ median age was 23 years (range 21–25), that of the professional translators was 37 years (range 25–51).

All participants had normal or corrected to normal vision. Two students wore contact lenses and one student wore glasses, yet the calibration with the eye tracker was successful for all three. Two professional translators wore lenses. Calibration was problematic for one of the professionals, their sessions with problematic calibration were removed from the data.

The project this study is a part of has been reviewed and approved by the Ethical Committee of the Faculty of Psychology and Educational Sciences at Ghent University. All participants gave written informed consent.

Students reported that they were aware of the existence of MT systems, and sometimes used them as an additional resource, but they had received no explicit post-editing training. Some professional translators had basic experience with post-editing, although none of the translators had ever post-edited an entire text.

All participants performed a LexTALE test (Lemhöfer and Broersma, 2012), which is a word recognition test used in psycholinguistic experiments, so as to assess English proficiency. Other than being an indicator of vocabulary knowledge, it is also an indicator of general English proficiency. As such, it is an important factor to take into account when comparing the translation process of students and professionals. Both groups had very high scores on this test; their scores (professionals mean = 88.27; standard deviation = 9.5; students mean = 88; standard deviation = 7.75) did not differ significantly [*t*(21) = 0.089, *p* = 0.47].

### Text Selection

Both to avoid specialized text experience playing a role and to control for text complexity as much as possible, 15 newspaper articles with a comparable complexity level were selected from Newsela, a website that offers English newspaper articles at various levels of complexity (as represented by Lexile^®^ scores, which combine syntactic and semantic information). We selected 150/160-word passages from articles with high Lexile^®^ scores (between 1160L and 1190L). To control texts further, we manually compared them for readability, potential translation problems and MT quality. Texts with on average fewer than 15 or more than 20 words per sentence were discarded, as well as texts that contained too many or too few complex compounds, idiomatic expressions, infrequent words or polysemous words. The Dutch MT output was taken from Google Translate (output obtained January 24, 2014), and annotated with a two-step Translation Quality Assessment approach^2^ (Daems et al., 2013, see also “MT Output Error Analysis”).

We discarded the texts for which the MT output would be too problematic, or not problematic enough, for post-editors, based on the number of structural grammatical problems, lexical issues, logical problems and mistranslated polysemous words. The final corpus consisted of eight newspaper articles, each 7–10 sentences long. The topics of the texts varied, and the texts required no specialist knowledge to be translated.

### MT Output Error Analysis

#### Annotation of the MT Output

To be able to identify the relationship between specific machine translation problems and post-editing effort, two of the authors annotated all translations for quality. Machine translation quality was analyzed from two perspectives.

The first was acceptability, or the adherence to target text and target language norms. This category contained all translation problems that can be identified by looking at the target text (in this case, the machine translation output) without consulting the source text. It consisted of the subcategories of grammar and syntax, lexicon, coherence, style and register, and spelling, which were all further subdivided into subcategories.

The second perspective was adequacy, or the adherence to source text norms. All problems that could be identified by comparing the source text with the target text belonged to this category. Subcategories included deletions and additions (of words or phrases), various types of mistranslations, and meaning shifts.

Each error type was given an error weight ranging from 0 (not a real error, but a change nonetheless, such as an explicitation) to 4 (contradictions and other problems that severely impacted the comprehensibility of the text).

The actual annotation took place in two steps, respecting both perspectives above: in step one the annotators were only given the target text and annotated the text for acceptability, and in step two annotators compared the translations with the source texts and annotated the texts for adequacy. The annotations of both annotators were then compared and discussed and only the annotations which both annotators agreed on were maintained for the final analysis. Annotations were made with the brat rapid annotation tool (Stenetorp et al., 2012).

#### Overview of Errors in the MT Output

**FIGURE 1:**
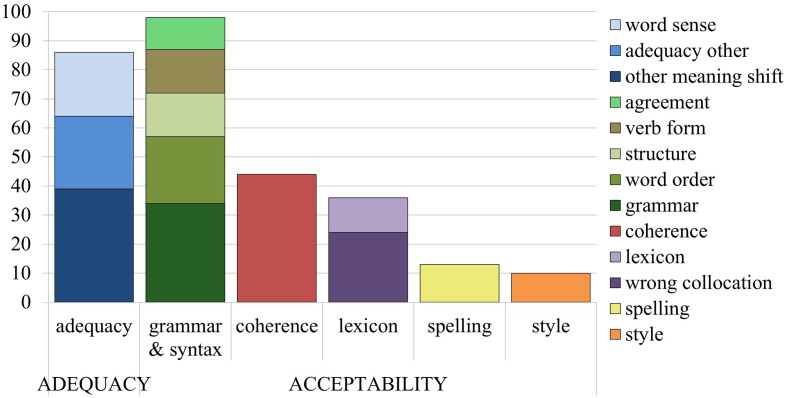
Error type frequency in the MT output.

Out of 63 sentences, 60 sentences contained at least one error. There were more acceptability issues (201 instances) than adequacy issues (86 instances). The error categorization described above contained 35 types of acceptability issues and 17 types of adequacy issues, but not all issues were found in the machine translation output^3^. To be able to perform statistical analyses, some of the error categories were grouped together, so that each error type occurred at least 10 times. The final classification used in this study can be seen in **Figure 1**.

From the perspective of ‘acceptability,’ it was the grammar and syntax category, which turned out to be the most common error category for MT output, with word order issues, structural issues, and incorrect verb forms occurring more than 10 times each. The different types of agreement issues (noun-adjective, article-noun, subject-verb, and reference) were grouped into a new ‘agreement’ category, and the other grammatical issues were contained in a ‘grammar other’ category (superfluous or missing elements). For coherence issues, the category of ‘logical problem’ occurred more than 10 times, but the other categories together (conjunction, missing info, paragraph, and inconsistency) did not occur more than 10 times, so all other coherence categories were grouped together. As for the lexicon, the subcategory of ‘wrong collocation’ appeared often enough to stand alone, while the other subcategories (wrong preposition, named entity, and word non-existent) were grouped into ‘lexicon other.’ All subcategories for style and spelling were merged together into the two main categories, since there were very few instances of these errors.

From the perspective of ‘adequacy,’ other meaning shifts and word sense issues^4^ occurred frequently enough to be considered as separate categories, while the other subcategories (additions, deletions, misplaced words, function words, part of speech, and inconsistent terminology) were grouped together into ‘adequacy other.’

The most common errors overall are grammatical errors (grammar and syntax), followed closely by adequacy issues.

### Procedure

#### Sessions

The data were gathered during two separate sessions for each participant. The students’ sessions took place in June and July 2014 and the professionals’ sessions took place in April and May 2015.

The first session started with a survey, to gain an idea of participants’ backgrounds, their experience with and attitude toward MT and post-editing, and a LexTALE test. This was followed by a copy task (participants copied a text to get used to the keyboard and screen) and a warm-up task combining post-editing and human translation, so that participants could get used to the environment and the different types of tasks. The actual experiment consisted of two texts that they translated from scratch, and two texts that they post-edited.

The second session started with a warm-up task as well, followed first by post-editing two texts and translating two texts from scratch. The final part of the session consisted of unsupervised retrospection (participants received the texts which they had just translated and were requested to highlight elements they found particularly difficult to translate) and another survey, to obtain insight into participants’ attitude after the experiment.

The order of the texts and tasks was balanced across participants within each group in a Latin square design. Participants were instructed to deliver products of publishable quality, whether they were translating from scratch or post-editing. There was no time limit, and participants could take breaks between texts if so requested, to mimic a natural translation day. All participants had a morning and an afternoon session, though temperature and light conditions remained constant throughout the different sessions, as they took place in a basement room (controlled light is needed for accurate eyetracking data).

#### Registration Tools

To be able to study all aspects of the translation process, the process was registered with two keystroke logging tools and an EyeLink 1000 eyetracker.

The main keystroke logging tool was the CASMACAT translators’ workbench (Alabau et al., 2013), which is an actual workbench with added mouse tracking and keystroke logging software. A browser plugin directly added the EyeLink fixation data to the CASMACAT logging files. Though not relevant for this particular analysis, we also used the Inputlog keystroke logging tool (Leijten and Van Waes, 2013) to register the usage of external resources during translation and post-editing.

The texts were presented to the participants one by one, with eye tracker calibration taking place before each new text. Only one sentence could be edited at a time, although the entire text was visible and participants could go back and forth through the text. The source text was shown on the left hand side of the screen, the right hand side contained the MT output.

### Analysis

The final dataset comprised 721 post-edited sentences, concatenated from all post-editing sessions and enriched with MT quality information. For each sentence, the average error weight per word was calculated by summing up the error weight of all errors found in the sentence and dividing that value by the number of words in the sentence.

The statistical software package R (R Core Team, 2014) was used to analyze the data. We used the lme4 package (Bates et al., 2014) and the lmerTest package (Kuznetsova et al., 2014-48pc) to perform linear mixed effects analyses. In these analyses, statistical models can be built that contain independent variables as well as random effects, i.e., subject or item-specific effects. It is also possible to have more than one independent variable, and to study the interaction effect of independent variables on one another. A statistical model is always tested against a null model, i.e., a model that assumes that there is no effect on the dependent variable. If the created model differs sufficiently from the null model, we can assume that there is an effect from the independent variables on the dependent variables.

In this particular study, we used the various post-editing effort indicators as dependent variables, see **Table 1** for an overview of dependent variables with descriptives for student and professional data; The independent variables are machine translation quality and experience. They were included in the models with interaction effect, as we are interested in seeing how machine translation quality has a different influence on effort indicators for students and professional translators. The random effects were participants and sentence codes (a unique id for each source text sentence), because we expected there to be individual differences across participants as well as sentence-inherent effects.

**Table 1:** Overview of dependent variables.

Variable (measure)		*M*	Median	*SD*	*SE*	min	max
Average duration per word (Time in ms divided by the number of words in the sentence)	Professional	4560	3726	4088	207	0	41986
	Student	4750	3573	3573	197	0	26553
	
Average fixation duration (Total fixation time in ms divided by the number of fixations in the sentence)	Professional	241	243	34	1.72	145	342
	Student	244	238	57	3.13	0	513
	
Average number of fixations (Total number of fixations divided by the number of words in the sentence)	Professional	18.27	16	10.46	0.53	8.07	94.5
	Student	17/0.77	15.91	10.13	0.56	0	68.1
	
Average number of production units (Total number of production units divided by the number of words in the sentence)	Professional	0.41	0.38	0.24	0.01	0	1.42
	Student	0.43	0.42	0.26	0.01	0	1.36
	
Pause ratio [Total time in pauses (in ms) divided by total editing time (in ms)]	Professional	0.78	0.86	0.23	0.01	0	1
	Student	0.85	0.87	0.12	0.01	0	1
	
Average pause ratio [Average time per pause (in ms) in a sentence divided by the average time per word (in ms) in the sentence]	Professional	2.48	1.91	2.55	0.13	0	22.9
	Student	2.93	1.85	3.2	0.18	0	24.33
	
Human-targeted translation error rate (HTER) (Edit distance between machine translation output and the post-edited sentence)	Professional	53.49	54.55	24.56	1.24	0	117
	Student	51.62	51.85	23.54	1.3	0	113


For each of the models discussed below, we started by building a null model with the post-editing effort indicator as dependent variable and participant and sentence code as random effects. This model was then tested against the model containing two predictors (the continuous variable machine translation quality and the categorical variable experience, which was either ‘student’ or ‘professional’), plus interaction effect.

As a measure of model fit, we used Akaike’s Information Criterion (AIC) values (Akaike, 1974). The model with the lower AIC value has a better fit compared to the model with the higher AIC value, especially when the difference between both models is greater than 4 (Burnham and Anderson, 2004), but an AIC value in itself has no absolute meaning, which is why we only included difference in AIC value in the tables.

We looked at the impact of fine- and coarse-grained machine translation quality on different post-editing effort indicators in two different analyses. This was done to first establish whether effort indicators are indeed impacted by machine translation errors, and to verify whether the suggestion by Koponen et al. (2012) and Stymne et al. (2012) to use more fine-grained error typologies really leads to more obvious differences between the different post-editing effort indicators.

The first analysis was the most coarse-grained, in which we used the average total machine translation error weight and experience as predictor variables. For each of the post-editing effort indicators, we built a null model and a model with both predictor variables, which we then compared.

For the fine-grained analysis, we also built separate models for each of the post-editing effort indicators. Here, we added all our adapted machine translation error subcategories as possible predictors. We then tested which of the subcategories were retained by the step-function from the lmerTest package. For each of these significant predictors, we built a separate model, adding experience with interaction effect as a possible additional predictor.

## Results

### Analysis 1: Coarse-Grained

A summary of the models with average total machine translation error weight and experience plus interaction as possible predictor variables can be found in **Table 2**. The table contains the dependent variable, i.e., the post-editing effort indicator under scrutiny in the first column, the difference in AIC value between the predictor model and the null model (i.e., model without predictor) in the second column, an overview of the significant or almost significant predictors (i.e., MT error weight and/or experience, with or without interaction effect, with *p* ≤ 0.05) in the third column, the effect size for each significant predictor in column four, and the *p*-value to indicate significance in column five. The effect column shows the actual effect that an independent variable has on a dependent variable. For non-significant effects, the table contains ‘n/a.’

**Table 2:** Summary of mixed models with average total MT error weight and experience plus interaction effect as fixed effects.

Dependent variable	Difference in AIC value	Significant predictors	Effect	*p*
Average duration per word (in ms)	-5	MT error weight	3526 (±1224)	0.005
Average fixation duration (in ms)	2	n/a	n/a	n/a
Average number of fixations	-7	MT error weight	10 (±2.7)	<0.001
Average number of production units	-20	MT error weight	0.3 (±0.06)	<0.001
Pause ratio	-10	Experience	0.1 (±0.03)	0.002
Average pause ratio	-17	MT error weight	-2.33 (±0.75)	0.002
		Experience	1.06 (±0.53)	0.05
HTER	-10	MT error weight	31 (±8)	<0.001


**FIGURE 2:**
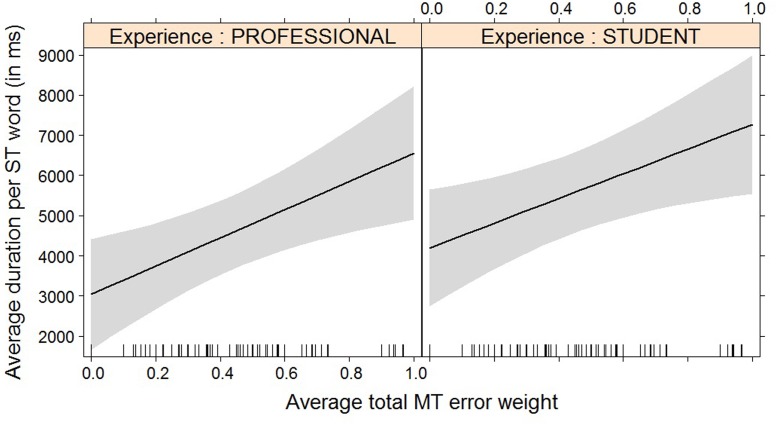
Effect plot of total MT error weight on average duration. An increase in average total MT error weight leads to an increase in average duration per ST word, for professionals and students alike.

**FIGURE 3:**
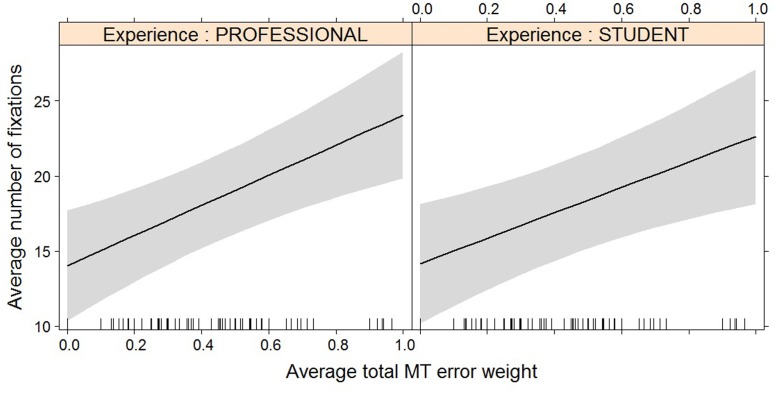
Effect plot of total MT error weight on average number of fixations. An increase in average total MT error weight leads to an increase in average number of fixations, for professionals and students alike.

**FIGURE 4:**
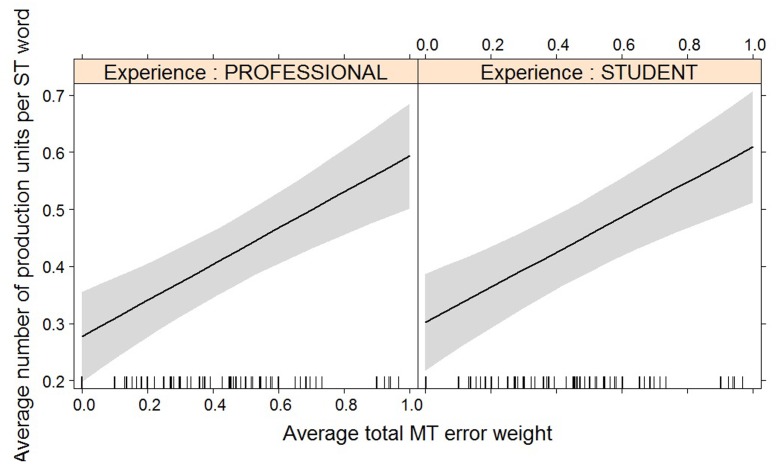
Effect plot of total MT error weight on average number of production units. An increase in average total MT error weight leads to an increase in average number of production units, for professionals and students alike.

**FIGURE 5:**
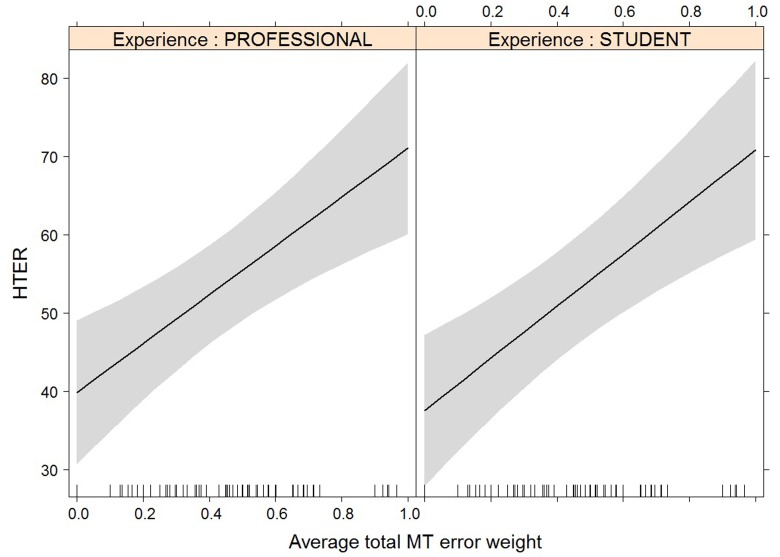
Effect plot of total MT error weight on human-targeted translation error rate (HTER). An increase in average total MT error weight leads to an increase in HTER score, for professionals and students alike.

**FIGURE 6:**
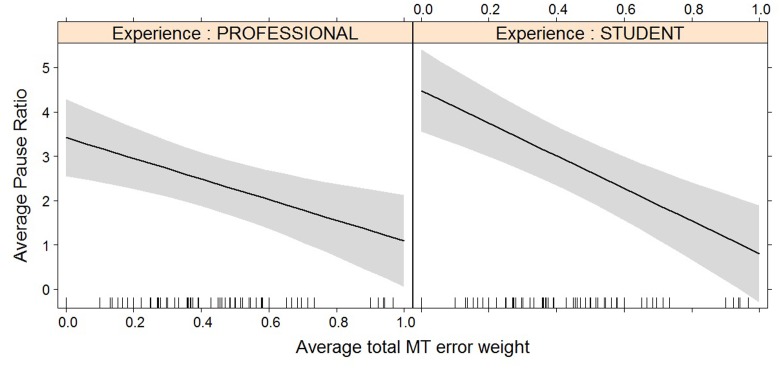
Effect plot of total MT error weight on average pause ratio. An increase in average total MT error weight leads to a decrease in average pause ratio, for professionals and students alike.

**FIGURE 7:**
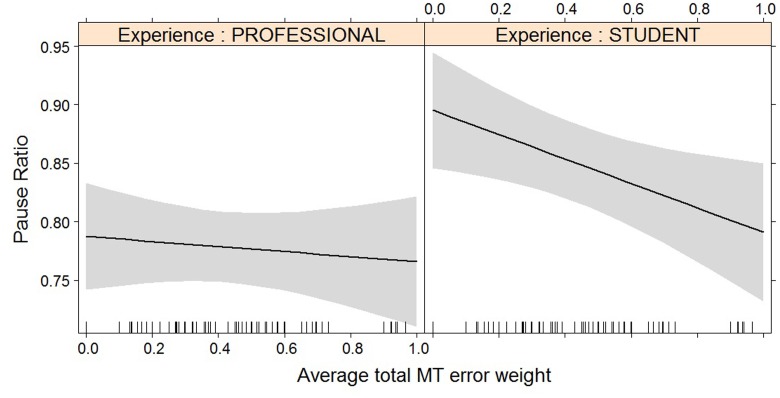
Effect plot of total MT error weight on pause ratio. Students show a higher average pause ratio than professional translators; the impact of the increased error weight on pause ratio is not statistically significant.

For most post-editing effort indicators, machine translation quality has an impact, but experience does not, with the exception of both pause measures. The effect of machine translation quality on the effort indicators is in line with expectations, with a decrease in quality leading to an increase in duration needed (**Figure 2**), an increase in the number of fixations (**Figure 3**) and the number of production units (**Figure 4**), an increase in HTER score (**Figure 5**), and a decrease in average pause ratio (**Figure 6**). The post-editing effort indicators on which machine translation quality seems to have no statistically significant impact are average fixation duration and pause ratio. The latter is impacted by experience rather than machine translation quality (**Figure 7**), whereas the average pause ratio is impacted by experience as well as machine translation quality. Students have a significantly higher pause ratio than professionals, presumably requiring more time to think before changing anything, but also a higher average pause ratio, which is somewhat surprising. It must be noted, however, that the latter effect is not quite significant (*p* = 0.05) and so it must be interpreted with caution. We further found no significant effect of either machine translation error weight or experience on average fixation duration.

### Analysis 2: Fine-Grained

We obtain a more nuanced picture when studying the results for the fine-grained level analysis in **Table 3**. We first built a model including all possible machine translation error types as possible predictors for the different post-editing effort indicators. The significant error types are listed in column two, showing that different types of machine translation errors impact different post-editing effort indicators. The more technical effort indicators (number of production units, pause ratio, average pause ratio, and HTER) are mostly impacted by grammatical errors (grammar, structure, word order, and agreement), whereas the more cognitive effort indicators (fixations and duration) are influenced most by coherence and other meaning shifts. We then built a model for each of the significant MT error types separately and added experience plus interaction effect as possible predictors. The significant (*p* < 0.05) and almost significant (*p* < 0.06) predictors for these models can be seen in column four, with the actual effect size in column five. For non-significant effects, the table contains ‘n/a.’

**Table 3:** Summary of mixed models with average MT error weight for the subcategories retained by step-function as fixed effects, and experience plus interaction effect as potential additional fixed effects.

Dependent variable	Predictor retained	Difference in AIC value	Significant predictors	Effect	*p*
Average duration per word (in ms)	Coherence	-10	MT error weight	9866 (±2642)	0.003
			Experience	1338 (±622)	0.041
			MT EW:Exp	-3799 (±1963)	0.054

Average fixation duration (in ms)	Other meaning shift	-9	MT EW:Exp	51.64 (±16.63)	0.002

Average number of fixations	Other meaning shift	-5	MT error weight	17.85 (±5.56)	0.002
	Coherence	-8	MT error weight	23 (±6)	0.002

Average number of production units	Other meaning shift	-4	n/a	n/a	n/a
	Grammar	1	n/a	n/a	n/a
	Structure	2	n/a	n/a	n/a
	Word order	1	MT error weight	0.43 (±0.22)	0.052

Pause ratio	Grammar	-10	Experience	0.08	0.002

Average pause ratio	Coherence	-4	n/a	n/a	n/a
	Structure	0	n/a	n/a	n/a

HTER	Agreement	-1	MT error weight	71 (±27)	0.01
	Other meaning shift	-6	MT error weight	39 (±17)	0.02
			MT EW:Exp	17.8 (±9)	0.053
	Spelling	-6	MT EW:Exp	147 (±62)	0.018


**FIGURE 8:**
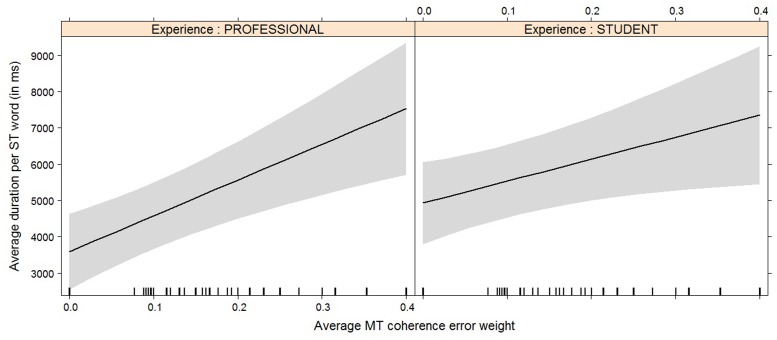
Effect plot of MT coherence error weight on average duration. An increase in average MT coherence error weight leads to an increase in the average duration. Students have a higher overall average duration, but the average duration increases somewhat more rapidly for professionals with an increase of MT coherence error weight than it does for students.

**FIGURE 9:**
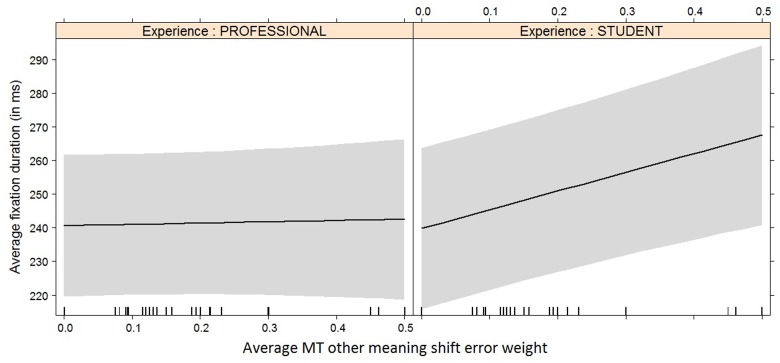
Effect plot of MT other meaning shift error weight on average fixation duration. Only the interaction effect is significant: the average fixation duration for students increases more heavily than that of professional translators with an increase in MT other meaning shift error weight.

**FIGURE 10:**
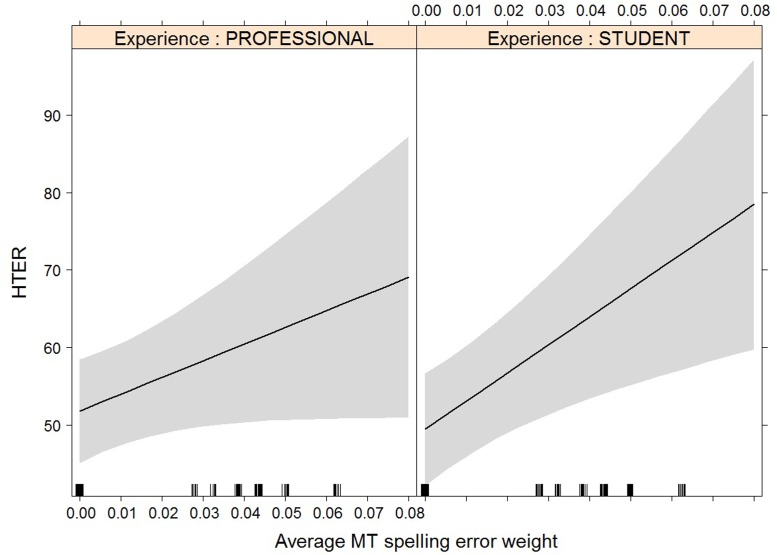
Effect plot of MT spelling error weight on HTER. Only the interaction effect is significant: the average HTER for students increases more heavily than that of professional translators with an increase in MT spelling error weight.

It is interesting to see how experience and/or experience with interaction effect now become relevant for the models with average duration (**Figure 8**), average fixation duration (**Figure 9**), and HTER (**Figure 10**) as dependent variables. In the case of average duration, students seem to be impacted less by an increase in coherence issues than professional translators, although the significance levels are not convincing (the experience effect only just reaches significance, the interaction effect almost reaches significance). In the case of average fixation duration, only students seem to be impacted by an increase in other meaning shifts, whereas the average fixation duration of professional translators remains comparable. The trend for HTER is similar, with students responding more strongly (yet not statistically significantly) to an increase in other meaning shifts than professional translators. In addition, HTER scores for students go up significantly with an increase in spelling errors. In the models for average number of production units and average pause ratio, a few different machine translation error types seem to influence the post-editing effort indicators, but once split into a separate model containing experience with interaction effect as additional predictors, the predictors are no longer significant. The model with word order as predictor variable of the average number of production units approaches significance, but that is the only one.

## Discussion

We expected to find that (i) post-editing effort indicators are influenced by machine translation quality, (ii) the product effort indicator HTER is influenced by other machine translation error types than process effort indicators, (iii) there is an overlap in error types that influence the various process effort indicators, and (iv) the effort indicators of student translators and professional translators respond to different error types in different ways. In order to verify these hypotheses, we studied the impact of machine translation quality on seven types of post-editing effort indicators (six process-based effort indicators and one product-based effort indicator), at two levels of MT quality assessment granularity and we added experience as a predictor.

### Hypothesis 1

From the coarse-grained analyses, we learned that post-editing effort indicators can indeed be predicted by machine translation quality, with the exception of average fixation duration and pause ratio.

Previous studies, too, have shown that average fixation duration does not differ significantly for good and bad MT quality (Doherty and O’Brien, 2009; Doherty et al., 2010), and so perhaps average fixation duration is not a good measure of post-editing effort.

For pause ratio, it seems that students require significantly more time in pauses than professional translators, and this effect outweighs the impact of machine translation quality. Perhaps students need more time to think about the correct course of action, whereas this process has been become more automatic for professionals. This confirms previous findings on students’ pause behavior (Dragsted, 2010), and might be an indication that pause ratio measures something else than average pause ratio (Lacruz et al., 2012), as the latter is influenced by machine translation quality.

### Hypothesis 2

Most importantly, the fine-grained analysis shows that two out of three predictors (agreement and spelling issues) of the product effort indicator HTER are unique predictors, i.e., they do not appear in the models for any of the other post-editing effort indicators. This seems to offer support for our hypothesis that product effort indicators measure different things than process effort indicators. On the other hand, the HTER predictor ‘other meaning shift’ is shared with a few process effort indicators as well, which means that HTER presumably does manage to measure some of the effort measured by process effort indicators.

### Hypothesis 3

Not all process effort indicators seem to be influenced by the same machine translation error types, refuting our third hypothesis. Duration, for example, is influenced most by coherence, whereas fixation duration is influenced by other meaning shifts. The machine translation error types that occur with more than one post-editing effort indicator are coherence issues, other meaning shifts, grammar and structural issues. As ‘other meaning shifts’ also occurred as a predictor of HTER, it would seem that being able to detect these three issue types (coherence issues, other meaning shifts, grammar and structural issues) would be the best way to ensure that as many different types of effort as possible are being taken into account.

The lack of word order issues as an influential category is striking, as we expected them to influence post-editing duration (Koponen et al., 2012), the average fixation duration, and number of fixations (Stymne et al., 2012). A possible explanation for these findings is the fact that our error classification includes error types such as coherence, which was not included in Temnikova’s (2010) classification, and those error types outweigh the effect of commonly used error types such as word order issues. Further research on a larger dataset and different languages is needed to support or refute these claims.

The more detailed findings can largely be explained by existing literature. The number of production units was influenced most by grammatical issues (Alves and Gonçalves, 2013), and fixations were influenced most by other meaning shifts (comparable to mistranslations), as suggested by Stymne et al. (2012). With respect to both pause measures, we found support for the claim made by Lacruz et al. (2012) that average pause ratio does not measure the same as pause ratio suggested by O’Brien (2006). Average pause ratio is influenced by structural issues and coherence issues, whereas pause ratio is influenced by grammatical issues, but this effect is outweighed by the influence of experience.

### Hypothesis 4

Regarding experience effects, we expected students to be more heavily influenced by grammatical and lexical issues and professional translators to be more heavily influenced by coherence and structural issues (Sommers, 1980; Hayes et al., 1987; Tirkkonen-Condit, 1990; Séguinot, 1991).

This almost holds true for the average duration per word, where an increase in coherence issues leads to an increase in the time needed and this effect is stronger for professional translators than for students. It must be noted, however, that this interaction effect is not quite significant (*p* = 0.054).

For the average fixation duration, which is influenced most heavily by other meaning shifts, only the interaction effect between the increase in other meaning shifts and experience is significant. While the average fixation duration for professional translators remains more or less constant as the number of other meaning shifts increases, the average fixation duration for students goes up under the same circumstances.

A comparable trend can be seen with HTER, although the effect here is not significant. This can be an indication that other meaning shifts are cognitively more demanding for students, seeing how it is not an issue they usually focus on during translation. For professional translators, however, spotting and solving these issues is probably more routinised, causing their average fixation duration to remain constant and their HTER scores to be lower (Tirkkonen-Condit, 1990; Séguinot, 1991). Students’ HTER scores were also more heavily influenced by spelling errors. This finding is somewhat harder to explain, as spelling issues are mostly related to compounds and spaces before punctuation, which are both error types that can be expected to be corrected by both students and professional translators. We would have to look at the final translations to see whether this was actually the case.

Experience had no effect on the number of fixations, production units, and average pause ratio, which could indicate that these three predictors measure a different kind of post-editing effort than the other predictors. These findings could be further evaluated by studying the final post-editing product and comparing the quality of student and professional post-editors’ texts.

### Limitations

Although we included data from students as well as professional translators, the total dataset remains relatively small. While our fine-grained analysis showed some promising results, it must be noted that with the more fine-grained analysis, fewer observations are taken into account, and so these results need to be interpreted with caution. Further experiments with the same categorisation on more data and different language pairs should be carried out in order to further develop the claims made in this article.

After this paper was written, Google released its neural machine translation for the English-Dutch language pair^5^. Future experiments will repeat the above analyses with this newer type of machine translation, to see whether the results can be replicated or to discover the ways in which neural machine translation differs from statistical machine translation with regards to its impact on post-editing effort.

## Conclusion

We confirmed that machine translation quality has an impact on product and process post-editing effort indicators alike, with the exception of average fixation duration and pause ratio.

While most machine translation error types occur with more than one post-editing effort indicator, two of HTER’s predictors are not shared with any of the other effort indicators, providing some support for our hypothesis that product effort measures do not necessarily measure post-editing effort the way process effort measures do. On the other hand, the various process effort indicators are influenced by different error types. This indicates that the question of what influences post-editing effort depends greatly on which type of effort is meant, with coherence issues, grammatical issues and other meaning shifts being good candidates for effort prediction on the basis of MT quality in the future. This also means that, depending on the type of effort one wishes to reduce, different error type predictors should be used. For example, a language service provider mostly concerned with translation speed should focus on coherence issues, as these had the greatest impact on translation duration.

In contrast with our expectations, experience only had a significant effect on four out of seven effort predictors: average duration per word, average fixation duration with an increase of other meaning shifts, pause ratio, and HTER with an increase of spelling issues. This either means that the other three effort indicators (number of fixations, average pause ratio, and number of production units) measure different types of effort, or that the differences between students and professional translators is smaller than often thought.

Once our determined important error types and their impact have been confirmed in larger studies, this information can – in future work – be used to improve translation tools, by only providing MT output to a translator when the effort to post-edit a sentence is expected to be lower than the effort to translate the sentence from scratch, and by taking into account that post-editor’s level of experience. Additionally, translator training (O’Brien, 2002) that incorporates post-editing can be adapted to make future translators more aware of effortful machine translation errors. By learning how to spot and solve these types of issues, the post-editing process can, in turn, become less strenuous as well.

## Author Contributions

JD, LM, SV, and RH discussed and agreed upon the design of the study and analysis. JD and LM performed the text selection and error analysis. LM provided the scripts for the HTER calculation. JD performed the original experiments and statistical analyses. RH offered suggestions related to data collection and improvements of the statistical analysis JD wrote the first draft of the article. LM, SV, and RH provided suggestions for revision. All authors approve the submission of the manuscript in the current state and agree to be accountable for all aspects of the work in ensuring that questions related to the accuracy or integrity of any part of the work are appropriately investigated and resolved.

## Conflict of Interest Statement

The authors declare that the research was conducted in the absence of any commercial or financial relationships that could be construed as a potential conflict of interest.

## Footnotes

1 Given that Koponen et al. (2012) found that keystrokes were more influenced by personal differences rather than differences in effort, we decided to include production units rather than keystrokes. Production units are editing events, i.e., continuous typing activities, that are often defined as units separated from one another by pauses of at least one second (Carl et al., 2016).

2 Instructions can be found online: users.ugent.be/∼jvdaems/TQA_guidelines_2.0.html

3 Issues not found in MT output: spelling: capitalization, typo; coherence: inconsistency, paragraph, coherence other; grammar: comparative/superlative, singular/plural; style: long/short sentences, text type; adequacy: contradiction, explicitation, hyperonymy, hyponymy, terminology, time.

4 Other meaning shifts are adequacy issues that cannot be classified as belonging to one of the other subcategories, and a word sense issue occurs when the wrong meaning of the word has been chosen for the context. For example, the phrase ‘donned shades,’ i.e., ‘put on sunglasses,’ was translated into Dutch as ‘vormden schaduwen’ (created shadows). The translation for ‘donned’ is a case of ‘other meaning shift,’ the translation for ‘shades’ is a word sense issue, ‘shadow’ being a possible translation for ‘shade,’ however, not in this context.

5 https://nederland.googleblog.com/2017/04/grote-verbetering-voor-het-nederlands.html?m=1
